# K‐medoids clustering of hospital admission characteristics to classify severity of influenza virus infection

**DOI:** 10.1111/irv.13120

**Published:** 2023-03-07

**Authors:** Aleda M. Leis, Erin McSpadden, Hannah E. Segaloff, Adam S. Lauring, Caroline Cheng, Joshua G. Petrie, Lois E. Lamerato, Manish Patel, Brendan Flannery, Jill Ferdinands, Carrie A. Karvonen‐Gutierrez, Arnold Monto, Emily T. Martin

**Affiliations:** ^1^ Department of Epidemiology University of Michigan School of Public Health Ann Arbor Michigan USA; ^2^ Epidemic Intelligence Service CDC Atlanta Georgia USA; ^3^ Wisconsin Department of Health Services Madison Wisconsin USA; ^4^ Departments of Internal Medicine and Microbiology and Immunology University of Michigan Ann Arbor Michigan USA; ^5^ Marshfield Clinic Research Institute Marshfield Wisconsin USA; ^6^ Department of Public Health Sciences Henry Ford Health System Detroit Michigan USA; ^7^ Influenza Division Centers for Disease Control and Prevention Atlanta Georgia USA

**Keywords:** disease severity, influenza, in‐hospital outcomes, k‐medoids clustering

## Abstract

**Background:**

Patients are admitted to the hospital for respiratory illness at different stages of their disease course. It is important to appropriately analyse this heterogeneity in surveillance data to accurately measure disease severity among those hospitalized. The purpose of this study was to determine if unique baseline clusters of influenza patients exist and to examine the association between cluster membership and in‐hospital outcomes.

**Methods:**

Patients hospitalized with influenza at two hospitals in Southeast Michigan during the 2017/2018 (n = 242) and 2018/2019 (n = 115) influenza seasons were included. Physiologic and laboratory variables were collected for the first 24 h of the hospital stay. K‐medoids clustering was used to determine groups of individuals based on these values. Multivariable linear regression or Firth's logistic regression were used to examine the association between cluster membership and clinical outcomes.

**Results:**

Three clusters were selected for 2017/2018, mainly differentiated by blood glucose level. After adjustment, those in C_17_1 had 5.6 times the odds of mechanical ventilator use than those in C_17_2 (95% CI: 1.49, 21.1) and a significantly longer mean hospital length of stay than those in both C_17_2 (mean 1.5 days longer, 95% CI: 0.2, 2.7) and C_17_3 (mean 1.4 days longer, 95% CI: 0.3, 2.5). Similar results were seen between the two clusters selected for 2018/2019.

**Conclusion:**

In this study of hospitalized influenza patients, we show that distinct clusters with higher disease acuity can be identified and could be targeted for evaluations of vaccine and influenza antiviral effectiveness against disease attenuation. The association of higher disease acuity with glucose level merits evaluation.

## 1 INTRODUCTION

Infectious respiratory diseases caused by influenza virus, respiratory syncytial virus, and SARS‐CoV‐2 can cause significant illness and are responsible for hundreds of thousands of hospitalizations in the United States annually.^1^ Data on in‐hospital progression of disease and treatment course are broadly available and used to evaluate severity of illness,^2^, ^3^ or the impact of vaccination^4^, ^5^ and treatment.^6^, ^7^, ^8^ However, the primary cause of admission, particularly in those with baseline multimorbidity, might be due to causes either exacerbated by milder respiratory tract infection (e.g., asthma) or possibly unrelated to infection (e.g., dehydration) rather than acute illness. This might bias results of vaccine or antiviral effectiveness against prevention or attenuation of severe disease. Differences in general health and health care seeking behaviour are difficult to directly measure,^9^, ^10^ and individuals may present and be admitted to the hospital at different stages in their disease course with varying disease severity. These patterns vary by population, health system, and specific aetiology.^11^, ^12^, ^13^, ^14^ While patients hospitalized with respiratory diseases such as influenza have historically been older with significant comorbidity,^11^, ^15^ the pattern has differed in various phases of the COVID‐19 pandemic.^16^

The heterogeneity of the hospitalized population at admission creates challenges when examining events occurring during hospitalization. Differential baseline comorbidity and presenting symptomology can significantly confound hospital data used as a surveillance metric for respiratory disease severity and can bias estimates of the effectiveness of interventions to reduce influenza morbidity or progression of disease.

Unsupervised machine learning algorithms provide a way to derive and characterize different groups of patients independent of an outcomes or treatment framework.^17^, ^18^ When applied to clinical data, this methodology can help identify distinct phenotypes of individuals driven by underlying relationships between health metrics. The aims of the current study were to develop clinically distinct clusters of patients based on laboratory and physiologic measurements within the first 24 h of hospitalization, to determine if cluster membership was associated with worse in‐hospital outcomes, and to evaluate the association of influenza vaccination on in‐hospital outcomes within a given cluster.

## 2 METHODS

Institutional review board approval for the US Hospitalized Adult Influenza Vaccine Effectiveness Network (HAIVEN) study was obtained from the University of Michigan. Cases were identified from 2017 to 2018 and 2018 to 2019 enrolees from a single site of the HAIVEN study, encompassing two major hospital systems in southeast Michigan (Michigan Medicine Hospital, Ann Arbor and Henry Ford Hospital, Detroit). Inclusion criteria for the HAIVEN cohort have been described elsewhere.^15^, ^19^ Briefly, adult patients ≥18 years of age were eligible for participation if presenting to the hospital within 10 days of symptom onset or worsening with a diagnosis or chief complaint broadly consistent with an acute respiratory illness such as influenza or pneumonia. Patients were prospectively recruited and completed a brief interview and research‐assistant performed specimen collection to determine laboratory‐confirmed influenza illness. Only individuals who tested positive for influenza were included in our analysis.

Participants were interviewed in‐person at the time of study enrolment. Data obtained from the electronic health record (EHR) via trained research staff chart review or database queries included demographics and comorbid conditions, acute illness characteristics such as symptom duration/type, laboratory and physiologic measures, and outcomes including ICU admission and hospital length of stay.^15^, ^19^

### 2.1 Physiologic characteristics of interest

Minimum and maximum values for physiologic and laboratory variables of interest were collected from the EHR for the first 24 h of hospitalization. Physiologic data included heart rate, respiratory rate, systolic blood pressure (SBP), temperature, and oxygen saturation.^20^ Laboratory data included non‐fasting blood glucose, haematocrit, haemoglobin, blood urea nitrogen (BUN), sodium, pH, total white blood cell (WBC) count, creatinine, platelets, bilirubin, and lactic acid.^20^ Estimated glomerular filtration rate (eGFR) was computed using the maximum creatinine value within the 24‐h window.^21^

### 2.2 Clustering of data

Variables were selected for clustering algorithm inclusion based on clinical relevance to indicating illness severity. Specific variables selected were as follows. Both minimum and maximum values were included unless otherwise specified: Temperature, heart rate (maximum), SBP, blood glucose, creatinine (maximum), haematocrit (minimum), sodium, WBC, platelets (minimum), respiratory rate (maximum), oxygen saturation (minimum), eGFR, and time from symptom onset to admission. Missing data for all selected physiologic measures were imputed using the study population mean stratified by age group (18–49, 50–64, 65+) and hospital. A table of selected metrics can be found in Table S1.

Prior to the creation of clusters, Hopkin's statistic was used to assess the randomness of the distribution of the data in relation to a uniform distribution. Values of .5 for this statistic indicate data are similar to the univariate distribution, while values closer to 1 indicate the data may contain clusters. The use of this statistic helps reduce the risk of a machine learning algorithm detecting clusters when the data do not actually have clusters within.^22^

Data were classified separately for each influenza season using the k‐medoids partitioning around the medoids (PAM) algorithm with Manhattan distance. Briefly, k‐medoids clustering assigns groups to a set of data based on the distance to an assigned central data point of a cluster.^23^ To start, these medoids are randomly assigned, and the algorithm iterates through selection of data centroids and cluster groupings until the distance from the centroid is minimized to all other data points in the cluster. K‐medoids clustering is more robust in the presence of outliers than other centroid‐based clustering algorithms such as k‐means because the chosen centroid is an observed data point. Additionally, this algorithm assigns all data observations to a cluster; this is preferred in a cohort of hospitalized individuals where biologically plausible data outliers are of interest. The appropriate number of clusters to be assigned for a given season was chosen using the largest average silhouette width, a measure of the distance from points in one cluster to another, with a maximum of 10 clusters tested.

The k‐medoids clustering was performed using the “pam” function in R. Following group assignment, the silhouette width of each cluster was computed using the “silhouette” function. An average silhouette width close to 1 indicates perfect clusters, and an average silhouette width around 0 indicates clusters lie close together. A negative silhouette width for a given observation indicates that the data point may have been misclassified.

### 2.3 Additional covariates

Additional covariates for adjusted analyses included age group (18–49, 50–64, 65+ years), sex, BMI, Charlson Comorbidity Index (CCI), admitting hospital, influenza strain and subtype, and influenza vaccination status.

### 2.4 Hospitalization severity metrics

Outcomes considered for severity of illness during the hospitalization included intensive care unit (ICU) admission, mechanical ventilator use, and total hospital length of stay (continuous, and prolonged defined as ≥8 days^24^).

### 2.5 Statistical analysis

General descriptive statistics were computed separately for each influenza season (2017/2018 and 2018/2019) and were reported as means with standard deviations, medians with interquartile range, or frequency and percentage, as appropriate. The normality of data and presence of outliers were assessed using histograms and box‐and‐whisker plots. Data clustering was performed as above within each year using the PAM algorithm. Characteristics between clusters were compared using Chi‐squared or Fisher's exact tests for categorical variables and independent *t* tests, ANOVA, Mann–Whitney *U*, or Kruskal–Wallis tests for continuous variables, as appropriate.

To determine if different classes of early hospitalization characteristics were associated with severe hospital sequelae, a series of models were constructed separately for each influenza season. For binary outcomes (ICU admission, ventilator use, and prolonged hospital length of stay), Firth's logistic regression models were constructed. Generalized linear models were used for the continuous outcome of hospital length of stay. Variables chosen a priori for model inclusion were k‐medoids cluster, age, sex, CCI (continuous), hospital, and influenza vaccination status. An exploratory analysis was conducted as above with the removal of outliers prior to clustering, with an outlier conservatively defined as a value <1st quartile‐1.5 * (interquartile range) or >3rd quartile + 1.5 * (interquartile range). Outliers were then imputed to the mean of remaining values stratified by age group and hospital. To maintain comparability with the primary analysis, the same number of clusters were implemented within a given influenza year.

Analysis was conducted using RStudio version 1.2.5042 and SAS v9.4 (SAS Institute, Cary, NC). A *P* value of .05 was considered statistically significant.

## 3 RESULTS

There were 242 individuals who met inclusion criteria from the 2017/2018 influenza season and 115 individuals from the 2018/2019 season (Table S2). Overall, patients were predominantly female, obese, and vaccinated against influenza. More individuals experienced ICU admission (13.0% vs. 6.2%; *P* = .031) and mechanical ventilation (16.5% vs. 9.9%; *P* = .074) in the 18/19 influenza season than the preceding season. Of those with a reported vaccination type, 56/147 (38.1%) in 17/18 and 19/73 (26.0%) in 18/19 received the trivalent vaccine. Overall characteristics of values included in the clustering algorithm are shown in Table 1.

**TABLE 1:** Patient characteristics included in k‐medoids algorithm overall by influenza season.

	2017/2018 season (N = 242)	2018/2019 season (N = 115)
**Clustering metrics measured within 24 H of admission**		
Time from symptom onset to admission (days)	2.4 (2.1)	2.6 (2.0)
Temperature		
Min	97.9 (0.6)	98.1 (0.6)
Max	100.0 (1.5)	100.1 (1.5)
Heart rate (max)	107.0 (18.3)	110.7 (19.2)
Systolic blood pressure		
Min	111.5 (16.7)	105.1 (15.6)
Max	137.0 (30.3)	126.3 (32.3)
Glucose		
Min	117.0 (49.1)	118.2 (56.3)
Max	159.5 (83.5)	175.0 (112.4)
Creatinine (max)	1.3 (1.6)	1.7 (2.1)
Haematocrit (min)	35.6 (5.4)	35.6 (6.2)
Sodium		
Min	135.7 (3.1)	135.3 (4.0)
Max	138.1 (3.3)	138.1 (3.9)
White blood cells		
Min	6.5 (3.7)	7.6 (8.4)
Max	8.6 (7.6)	10.2 (12.9)
Platelets (min)	178.6 (67.2)	193.8 (119.4)
Respiratory rate (max)	24.2 (5.5)	26.2 (8.2)
Oxygen saturation (min)	91.6 (4.4)	90.6 (5.0)
Estimated glomerular filtration rate	75.0 (39.8)	73.6 (40.8)

*Note*: Data are presented as mean (standard deviation).

### 3.1 2017/2018 cohort

**FIGURE 1:**
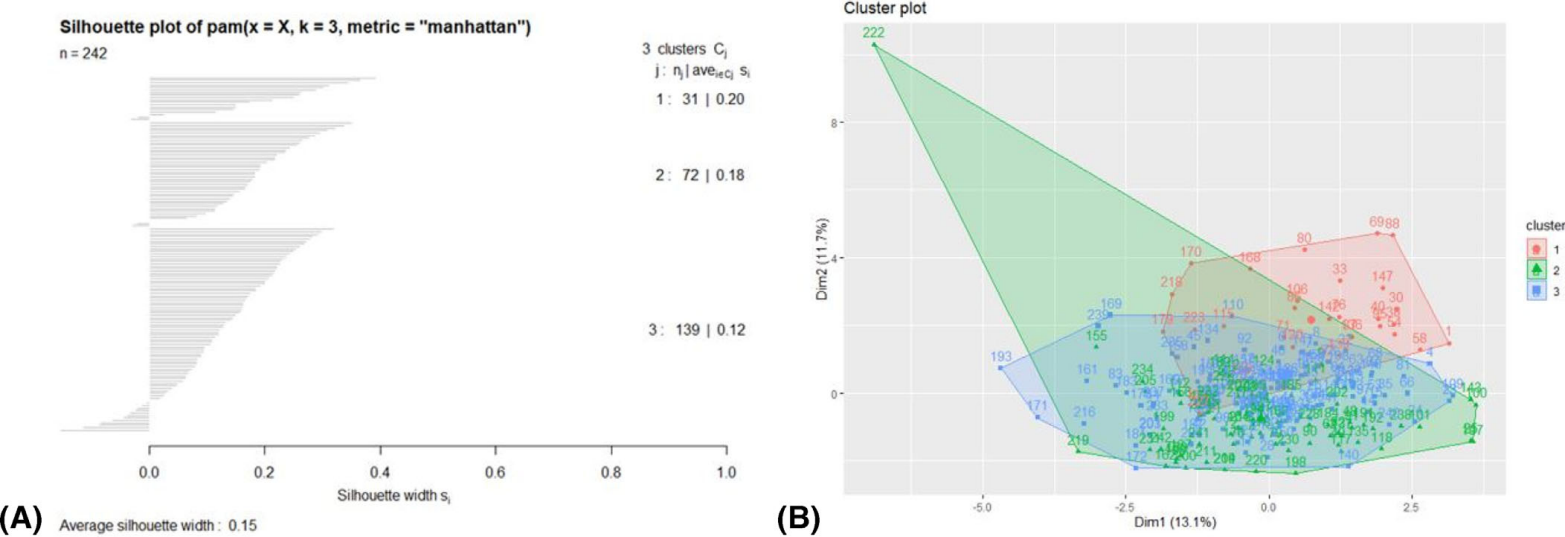
Clustering metrics for the 2017/2018 influenza season, including the silhouette plot of k‐medoids clusters (A) and the top two principal components of data in the k‐medoids clustering algorithm (B), with cluster membership highlighted.

The Hopkin's statistic for 2017/2018 was 0.810. The 3‐cluster model was selected with the highest average silhouette width of 0.15 (Figure 1). The silhouette plots indicate the possibility of minor misclassification of some individuals. There were significant differences in race, age, CCI, and diabetes between clusters (Table 2). For the variables included in the PAM algorithm, those in Cluster 1 for the 2017/2018 season (C_17_1) had significantly higher mean glucose (minimum mean 210.4 mg/dL, SD 66.9) than those in Cluster 2 (C_17_2) (minimum mean 90.5 mg/dL, SD 16.4) and Cluster 3 (C_17_3) (minimum mean 110.0 mg/dL, SD 26.7). Those in C_17_2 had significantly lower maximum heart rate, maximum systolic blood pressure, minimum white blood cell count, minimum platelets, and estimated glomerular filtration rate than the other two clusters. Those in C_17_2 also had significantly higher maximum creatinine than the other clusters. The rate of being mechanically ventilated was higher in C_17_1 than C_17_2 (22.6% vs. 6.9%), and the overall hospital length of stay was longer for those in C_17_1 than C_17_3 (mean 4.5 days [SD 4.4] vs. mean 2.8 days [SD 2.4]).

**TABLE 2:** Patient characteristics by k‐medoids cluster by influenza season.

	2017/2018 influenza season				2018/2019 influenza season		
	Cluster 1 (N = 31)	Cluster 2 (N = 72)	Cluster 3 (N = 139)	*P* value	Cluster 1 (N = 37)	Cluster 2 (N = 78)	*P* value
**Demographics**							
Age				.029^§^			.704
18‐49	6 (19.4)	15 (20.8)	44 (31.7)		14 (37.8)	24 (30.8)	
50–64	14 (45.2)	18 (25.0)	48 (34.5)		10 (27.0)	26 (33.3)	
≥65	11 (35.5)	39 (54.2)	47 (33.8)		13 (35.1)	28 (35.9)	
Male sex	10 (32.3)	35 (48.6)	54 (38.9)	.226	14 (37.8)	40 (51.3)	.177
Race							
White	10 (32.3)	51 (70.8)	48 (34.5)	<.001^†^, ^§^	19 (51.4)	33 (42.3)	.363
Black	18 (58.1)	20 (27.8)	79 (56.8)	<.001^†^, ^§^	18 (48.7)	42 (53.9)	.602
Asian	2 (6.5)	0 (0.0)	1 (0.7)	.018	0 (0.0)	1 (1.3)	.489
Hispanic	1 (3.2)	0 (0.0)	7 (5.0)	.152	0 (0.0)	5 (6.4)	.105
Hospital				<.001^†^, ^§^			.995
1	8 (25.8)	46 (63.9)	35 (25.2)		19 (51.4)	40 (51.3)	
2	23 (74.2)	26 (36.1)	104 (74.8)		18 (48.7)	38 (48.7)	
Flu strain and subtype/lineage				.852			.744
Type A—H1	1 (3.2)	4 (5.6)	10 (7.2)		15 (40.5)	29 (37.2)	
Type A—H3	22 (71.0)	41 (56.9)	86 (61.9)		14 (37.8)	33 (42.3)	
Type B—Victoria	0 (0.0)	0 (0.0)	2 (1.4)		0 (0.0)	2 (2.6)	
Type B—Yamagata	7 (22.6)	23 (31.9)	32 (23.0)		0 (0.0)	1 (1.3)	
Unknown subtype/lineage	1 (3.2)	4 (5.6)	8 (5.8)		8 (21.6)	13 (16.7)	
Unknown type	0 (0.0)	0 (0.0)	1 (0.7)		0 (0.0)	0 (0.0)	
Received influenza vaccine	17 (54.8)	48 (66.7)	82 (59.0)	.429	24 (64.9)	49 (62.8)	.832
**Comorbidities**							
Charlson Comorbidity Index, mean (SD)	3.9 (2.8)	4.3 (3.1)	2.4 (2.3)	<.001^‡^, ^§^	4.4 (2.7)	3.5 (3.0)	.139
Charlson Comorbidity Index Group				<.001^‡^, ^§^			.031
0	1 (3.2)	8 (11.1)	25 (18.0)		0 (0.0)	13 (16.7)	
1–2	11 (35.5)	16 (22.2)	64 (46.0)		13 (35.1)	22 (28.2)	
≥3	19 (61.3)	48 (66.7)	50 (36.0)		24 (64.9)	43 (55.1)	
BMI category				.353			.177
Underweight (<18.5)	0 (0.0)	2 (2.8)	5 (3.6)		1 (2.7)	4 (5.1)	
Normal/healthy weight (18.5–24.9)	3 (9.7)	14 (19.4)	34 (24.5)		7 (18.9)	17 (21.8)	
Overweight (25–29.9)	7 (22.6)	23 (31.9)	29 (20.9)		4 (10.8)	22 (28.2)	
Obese (30–39.9)	17 (54.8)	26 (36.1)	53 (38.1)		17 (46.0)	23 (29.5)	
Morbidly obese (≥40)	4 (12.9)	7 (9.7)	18 (13.0)		8 (21.6)	12 (15.4)	
High‐risk comorbidities							
Heart disease	14 (45.2)	45 (62.5)	50 (36.0)	.001^§^	23 (62.2)	38 (48.7)	.177
Heart failure	6 (19.4)	25 (34.7)	27 (19.4)	.039^§^	17 (46.0)	17 (21.8)	.008
Asthma	9 (29.0)	17 (23.6)	46 (33.1)	.359	10 (27.0)	17 (21.8)	.536
COPD	11 (35.5)	23 (31.9)	49 (35.3)	.881	16 (43.2)	28 (35.9)	.449
Other lung conditions	14 (45.2)	24 (33.3)	44 (31.7)	.354	21 (56.8)	35 (44.9)	.234
Diabetes	24 (77.4)	24 (33.3)	38 (27.3)	<.001^†^, ^‡^	29 (78.4)	25 (32.1)	<.001
Renal	13 (41.9)	42 (58.3)	36 (25.9)	<.001^§^	21 (56.8)	30 (38.5)	.065
Blood disorders	2 (6.5)	16 (22.2)	4 (2.9)	<.001^§^	6 (16.2)	9 (11.5)	.557
Immunosuppression	5 (16.1)	24 (33.3)	31 (22.3)	.104	10 (27.0)	25 (32.1)	.584
Malignancy	8 (25.8)	25 (34.7)	13 (9.4)	<.001^‡^, ^§^	7 (18.9)	22 (28.2)	.284
Metabolic disorders	20 (64.5)	39 (54.2)	49 (35.3)	.002^‡^, ^§^	20 (54.1)	43 (55.1)	.914
Liver disorders	3 (9.7)	11 (15.3)	14 (10.1)	.501	5 (13.5)	5 (6.4)	.288
Neurological/musculoskeletal	11 (35.5)	20 (27.8)	30 (21.6)	.228	11 (29.7)	26 (33.3)	.699
Cerebrovascular disorders	3 (9.7)	2 (2.8)	5 (3.6)	.242	1 (2.7)	3 (3.9)	.999
Endocrine	3 (9.7)	16 (22.2)	22 (15.8)	.258	8 (21.6)	13 (16.7)	.521
Long‐term medication	4 (12.9)	13 (18.1)	18 (13.0)	.586	8 (21.6)	17 (21.8)	.983
Morbid obesity	5 (16.1)	11 (15.3)	18 (13.0)	.844	12 (32.4)	12 (15.4)	.036
**Clustering metrics measured within 24 h of admission**							
Time from symptom onset to admission (days), mean (SD)	2.6 (2.1)	2.2 (2.0)	2.5 (2.0)	.597	2.6 (1.7)	2.6 (2.1)	.886
Temperature							
Min	97.8 (0.6)	97.9 (0.5)	97.9 (0.6)	.691	97.9 (0.5)	98.1 (0.6)	.036
Max	99.7 (1.5)	100.2 (1.5)	100.0 (1.5)	.190	99.9 (1.4)	100.2 (1.5)	.301
Heart rate (max), mean (SD)	109.1 (16.1)	101.3 (18.7)	109.5 (18.0)	.006^†^, ^§^	110.5 (20.6)	110.7 (18.7)	.966
Systolic blood pressure, mean (SD)							
Min	117.0 (15.2)	111.2 (17.0)	110.3 (16.7)	.134	104.4 (18.2)	105.4 (14.3)	.732
Max	149.5 (30.3)	126.5 (29.2)	139.7 (29.4)	.001^†^, ^§^	127.3 (38.9)	125.9 (28.9)	.828
Glucose, mean (SD)							
Min	210.4 (66.9)	90.5 (16.4)	110.0 (26.7)	<.001^†^, ^‡^, ^§^	157.2(82.2)	99.7 (21.4)	<.001
Max	311.6 (106.0)	139.4 (68.5)	136.0 (37.0)	<.001^†^, ^‡^	292.0 (133.4)	119.5 (26.3)	<.001
Creatinine (max), mean (SD)	1.2 (0.8)	1.8 (2.5)	1.2 (0.9)	.018^†^, ^§^	1.9 (2.4)	1.6 (2.0)	.502
Haematocrit (min), mean (SD)	36.7 (4.7)	34.6 (5.6)	36.0 (5.4)	.096	35.4 (6.2)	35.6 (6.2)	.885
Sodium, mean (SD)							
Min	134.4 (3.9)	136.0 (2.9)	135.9 (3.0)	.034^†^, ^‡^	134.0 (5.4)	135.9 (3.0)	.015
Max	137.1 (4.1)	139.3 (3.4)	137.7 (3.0)	.001^†^, ^§^	138.3 (4.7)	138.0 (3.5)	.754
White blood cells, mean (SD)							
Min	7.7 (3.4)	5.4 (5.0)	6.8 (2.7)	.005^†^, ^§^	8.3 (4.2)	7.2 (9.8)	.511
Max	9.4 (4.7)	8.9 (12.6)	8.3 (3.7)	.710	12.0 (7.1)	9.4 (14.9)	.317
Platelets (min), mean (SD)	171.5 (44.7)	121.3 (39.8)	209.9 (62.5)	<.001^†^, ^‡^, ^§^	190.9 (76.3)	195.2 (135.5)	.856
Respiratory rate (max), mean (SD)	25.9 (5.7)	23.8 (5.3)	24.0 (5.6)	.176	27.5 (9.3)	25.6 (7.7)	.243
Oxygen saturation (min), mean (SD)	90.7 (4.9)	91.1 (5.3)	92.0 (3.7)	.182	88.5 (6.7)	91.6 (3.7)	.002
Estimated glomerular filtration rate, mean (SD)	71.6 (26.2)	59.4 (28.2)	83.8 (44.7)	<.001^†^, ^§^	65.7 (40.8)	77.3 (40.6)	.154
**Outcomes**							
ICU Admission	3 (9.7)	4 (5.6)	8 (5.8)	.690	8 (21.6)	7 (9.0)	.060
Mechanical ventilator use	7 (22.6)	5 (6.9)	12 (8.6)	.038^†^	12 (32.4)	7 (9.0)	.002
Hospital LOS, mean (SD)	4.5 (4.4)	3.5 (2.9)	2.8 (2.4)	.009^‡^	5.3 (5.7)	2.8 (2.0)	.001
Prolonged LOS (≥8 days)	4 (12.9)	4 (5.6)	7 (5.0)	.250	6 (16.2)	3 (3.9)	.021

*Note*: Data are presented as column frequency (percentage) or mean (standard deviation [SD]). Overall *P* values were computed using chi‐square for categorical variables and ANOVA or Kruskal–Wallis for continuous variables. Missing data were imputed for cluster variables based on age‐ and hospital‐specific mean values.

^†^ 2017/2018 Cluster 1 versus 2017/2018 Cluster 2, *P* < .05.

^‡^ 2017/2018 Cluster 1 versus 2017/2018 Cluster 3, *P* < .05.

^§^ 2017/2018 Cluster 2 versus 2017/2018 Cluster 3, *P* < .05.

**FIGURE 2:**
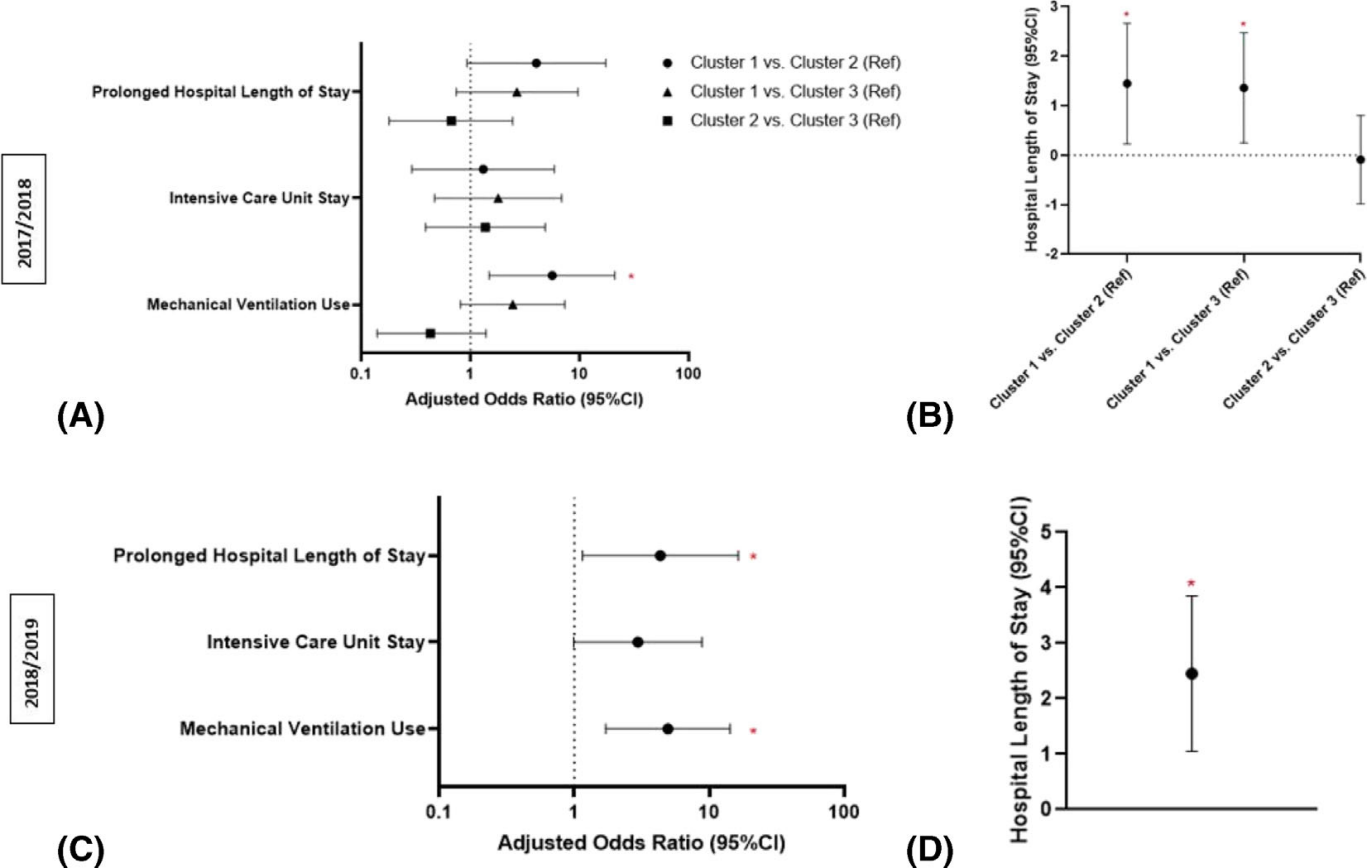
Adjusted odds ratios (A,C) and difference in model‐adjusted means (B,D) with 95% confidence intervals for outcomes. * indicates statistically significant differences between comparison groups. Models were adjusted for age, sex, hospital, continuous CCI, and influenza vaccination status.

After adjustment for age group, sex, hospital, continuous CCI, and influenza vaccination status, those in C_17_1 had 5.6 times the odds of having a mechanical ventilator than those in C_17_2 (95% CI: 1.49, 21.1; Figure 2). Additionally, those in C_17_1 had a significantly longer model‐adjusted mean hospital length of stay than those in both C_17_2 (mean 1.5 days longer, 95% CI: 0.2, 2.7) and C_17_3 (mean 1.4 days longer, 95% CI: 0.3, 2.5). There were no significant differences between clusters for the outcomes of ICU stay or prolonged hospital stay. Vaccination status was not associated with adverse outcomes in the fully adjusted models.

### 3.2 2018/2019 cohort

**FIGURE 3:**
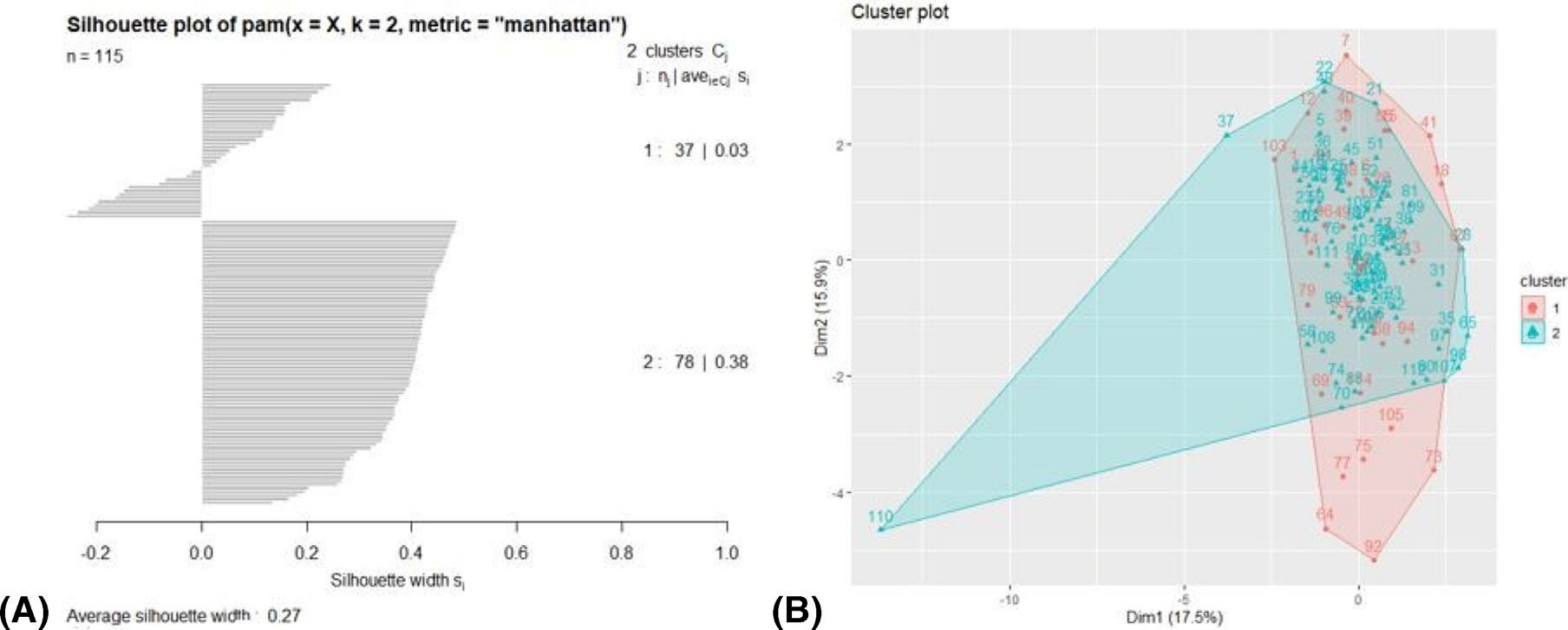
Clustering metrics for the 2018/2019 influenza season, including the silhouette plot of k‐medoids clusters (A) and the top two principal components of data in the k‐medoids clustering algorithm (B), with cluster membership highlighted.

The Hopkin's statistic for 2018/2019 was 0.837. The 2‐cluster model was selected with the highest average silhouette width of 0.27 (Figure 3). The silhouette plots indicate the possibility of moderate misclassification of some individuals in Cluster 1 for the 2018/2019 season (C_18_1). There were significant differences in underlying comorbidity between clusters, though there were no major differences in demographics (Table 2). For the variables included in the PAM algorithm, those in C_18_1 had significantly higher mean glucose (minimum mean 157.2 mg/dL, SD 82.2) than those in Cluster 2 (C_18_2) (minimum mean 99.7 mg/dL, SD 21.4). Additionally, those in C_18_1 had significantly lower minimum oxygen saturation than those in C_18_2 (mean 88.5 [SD 6.7] vs. mean 91.6 [SD 3.7]). The C_18_1 group had higher rates of mechanical ventilation (32.4% vs. 9.0%) and prolonged hospital stay ≥8 days (16.2% vs. 3.9%) than those in C_18_2, as well as a longer hospital stay (mean 5.3 days [SD 5.7] vs. mean 2.8 days [SD 2.0]).

After adjustment for age group, sex, hospital, continuous CCI, and influenza vaccination status, those in C_18_1 had 4.9 times the odds of being mechanically ventilated than those in C_18_2 (95% CI: 1.7, 14.3; Figure 2), and 4.3 times the odds of having a prolonged hospital stay (95% CI: 1.2, 16.4). After adjustment, those in C_18_1 had a significantly longer model‐adjusted mean hospital length of stay than those in C_18_2 (mean 2.5 days longer, 95% CI: 1.1, 3.9). Vaccination status was not associated with adverse outcomes in the fully adjusted models.

### 3.3 Sensitivity analysis

Imputation of outliers in both influenza cohorts and their effect on the sample are shown in Table S3. After model adjustment with the new clusters, there were no statistically significant differences in the odds or model‐adjusted means of outcomes for the 2017/2018 influenza season (Figure S1). For the 2018/2019 influenza season, after adjustment those in new cluster 1 had significantly higher odds of an intensive care unit stay compared with those in new cluster 2 (OR 4.62, 95% CI: 1.34, 15.97; Figure S1).

## 4 DISCUSSION

In this cohort of individuals in the 2017/2018 and 2018/2019 influenza seasons, we created clinically meaningful groups using k‐medoids clustering to improve the analysis of severity in a population of patients hospitalized with influenza. Our results suggest that those who were in clusters with hyperglycaemia and lower oxygen saturation at admission had higher risk of adverse in‐hospital sequelae and are thus potential cohorts of interest for further study of vaccine or antiviral effects.

We found glucose to be significantly different between clusters, with one cluster having significantly higher glucose in both years. The distribution of diabetes was also consistent across years, with approximately 70% prevalence in the high‐glucose clusters and 30% prevalence in the non‐hyperglycaemic clusters. Together, these results highlight that the use of simple dichotomous classifications for complex conditions such as diabetes may not accurately indicate a patient's risk for adverse outcomes. Indeed, controlling for such complex confounding has long been problematic within infectious disease severity research, most recently when examining treatments and hospital outcomes related to infection with SARS‐CoV‐2, leading to inconsistent results.^25^, ^26^, ^27^ This challenge is due in part to differential measurement and management of confounding, including analyses at the point of hospitalization admission, given model limitations in the number of confounders that can be included, and their often‐complex interrelationships. The use of techniques such as k‐medoids clustering to simultaneously account for multiple measures of comorbidity and group like patients together independent of outcomes‐based analysis provides a tool to increase homogeneity within groups and heterogeneity across groups for a more robust confounding adjustment.

More traditional dimensional reduction methods such as the use of propensity score matching have often been used to account for differential patterns of comorbidities between groups of interest. While propensity score matching is useful in reducing heterogeneity in the presence of a single exposure of interest, it becomes complex in instances where multiple treatments or exposures are being compared simultaneously. Additionally, there is inherent reduction in sample size when matching, limited by the number of individuals with and without the exposure having similar propensity scores; individuals in either group with uncommon comorbidity profiles may be overlooked and excluded from the matching if their propensity score does not align. For example, a 2020 study by Groeneveld et al examining the effective of oseltamivir lost 36% of oseltamivir patients and 65% of controls when matching, reducing the sample size to 88 pairs.^6^ While use of propensity score matching has been shown to reduce bias,^28^ such significant loss of data, especially in a rare‐outcomes setting, may lead to an increase in Type II error, and thus incorrect conclusions, due to inadequate power.^29^, ^30^ K‐medoids clustering can be used to identify subgroups that are biologically different without such restrictions, maintaining sample size for more robust analysis of effect modification by multiple treatment types. It should be noted that outliers within the range of biologically normal values are of great clinical significance, as these individuals may be at higher risk for adverse outcomes. K‐medoids clustering is robust to such outliers through use of data‐derived centroids for the clusters, rather than an arbitrary mean.

This study has several strengths, most notably that the cohort was nested within a large prospective two‐centre study of influenza vaccine effectiveness across multiple seasons, allowing for a robust and diverse analytic cohort. Both case definition and EHR data capture were standardized across sites, reducing heterogeneity of data quality. Additionally, the use of two hospitals within our region allowed for a more generalizable analysis. The biggest limitation of the study is small sample size and small number of outcomes, and due to missing data we were unable to adjust for vaccination type in modelling; however, we believe our analysis has minimized some of the bias from these limitations. Finally, while the k‐medoids clusters presented here may not generalizable to other cohorts, the methodology has many direct and current applications in severity analysis.

One of the most immediate applications can be for evaluating the effectiveness of new and existing antivirals for severe respiratory disease. Previous studies of such treatments have utilized traditional methods of covariate adjustment, which may contribute to heterogeneity of study findings.^31^ The use of this clustering method to phenotype baseline presentation can reduce this confounding and can be quickly implemented for these analyses. Such a technique will be needed as we continue understand how new antiviral treatments affect severity, and how vaccination impacts severity in instances of low vaccine effectiveness.

## 5 CONCLUSIONS

In conclusion, we found it was possible to cluster adult patients hospitalized with influenza into clinically distinct groups by baseline characteristics independent of a clinical outcome. Those with hyperglycaemia and lower oxygen saturation at admission were more likely to experience adverse events in our cohort, including prolonged hospitalization. The k‐medoids algorithm is a promising approach to disentangling the heterogeneity surrounding hospital admissions.

## AUTHOR CONTRIBUTION

**Aleda M. Leis**: Conceptualization (equal), formal analysis (lead), methodology (lead), software (lead), writing—original draft preparation (lead), writing—review and editing (equal). **Erin McSpadden**: Data curation (supporting), writing—review and editing (equal). **Hannah E. Segaloff**: Conceptualization (equal), methodology (supporting), writing—review and editing (equal). **Adam S. Lauring**: Data curation (supporting), writing—review and editing (equal). **Caroline Cheng**: Data curation (lead), formal analysis (supporting), software (supporting), writing—review and editing (equal). **Joshua G. Petrie**: Conceptualization (supporting), writing—review and editing (equal). **Lois E. Lamerato**: Data curation (supporting), writing—review and editing (equal). **Manish Patel**: Conceptualization (equal), methodology (supporting), writing—review and editing (equal). **Brendon Flannery**: Conceptualization (supporting), methodology (supporting), writing—review and editing (equal). **Jill Ferdinands**: Conceptualization (equal), data curation (supporting), writing—review and editing (equal). **Carrie A. Karvonen‐Gutierrez**: Conceptualization (supporting), supervision (supporting), writing—review and editing (equal). **Arnold Monto**: Conceptualization (supporting), writing—review and editing (equal). **Emily T. Martin**: Conceptualization (equal), funding acquisition (lead), methodology (supporting), supervision (lead), writing—original draft preparation (supporting), writing—review and editing (equal).

## CONFLICT OF INTEREST STATEMENT

ASL reports receiving research funding from the Centers for Disease Control and Prevention, National Institutes for Health, Burroughs Wellcome Fund, and FluLab, and receiving consulting fees from Sanofi (oseltamivir) and Roche (baloxavir) outside of submitted work. LEL reports receiving funding from the Centers for Disease Control and Prevention. AM reports receiving research funding from the Centers for Disease Control and Prevention. ETM reports receiving research funding from the Centers for Disease Control and Prevention and grant funding from Merck.

### PEER REVIEW

The peer review history for this article is available at https://publons.com/publon/10.1111/irv.13120.

## Supporting information


**Table S1.** Criteria for selection of laboratory and physiologic characteristics included in k‐medoids clustering algorithm.
**Table S2.** Patient characteristics overall by influenza season.
**Table S3.** Patient characteristics included in k‐medoids algorithm overall by influenza season for exploratory analysis with statistically defined outliers.
**Figure S1.** Adjusted odds ratios (A, C) and difference in model‐adjusted means (B, D) with 95% confidence intervals for outcomes. Models were adjusted for age group, sex, hospital, continuous CCI, and influenza vaccination status.

## Data Availability

Data are available upon reasonable request to study investigators.
