# Supplementary material for: K‐medoids clustering of hospital admission characteristics to classify severity of influenza virus infection
Source: Influenza Other Respir Viruses. 2023 Mar 7;17(3):e13120. doi: 10.1111/irv.13120 (PMC9992770; doi:10.1111/irv.13120)
Supplement: Supplementary file 1 — Table S1. Criteria for selection of laboratory and physiologic characteristics included in k‐medoids clustering algorithm. Table S2. Patient characteristics overall by influenza season. Table S3. Patient characteristics included in k‐medoids algorithm overall by influenza season for exploratory analysis with statistically defined outliers. Figure S1. Adjusted odds ratios (A, C) and difference in model‐adjusted means (B, D) with 95% confidence intervals for outcomes. Models were adjusted for age group, sex, hospital, continuous CCI, and influenza vaccination status. [file IRV-17-e13120-s001.docx]

**Table S1.** Criteria for selection of laboratory and physiologic characteristics included in k-medoids clustering algorithm.

| **Variable** | **Minimum Value Included** | **Maximum Value Included** |
| --- | --- | --- |
| Time from symptom onset to admission | NA | NA |
| Temperature | X | X |
| Heart Rate |  | X |
| Systolic Blood Pressure | X | X |
| Glucose | X | X |
| Creatinine |  | X |
| Hematocrit | X |  |
| Sodium | X | X |
| White Blood Cell Count | X | X |
| Platelets | X |  |
| Respiratory Rate |  | X |
| Oxygen Saturation | X |  |
| Estimated glomerular filtration rate | NA | NA |

**Table S2.** Patient characteristics overall by influenza season

|  | **2017/2018 Season**  **(N = 242)** | **2018/2019 Season**  **(N = 115)** |
| --- | --- | --- |
| **Demographics** | | |
| Age |  |  |
| 18-49 | 65 (26.9) | 38 (33.0) |
| 50-64 | 80 (33.1) | 36 (31.3) |
| ≥ 65 | 97 (40.1) | 41 (35.7) |
| Male Sex | 99 (40.9) | 54 (47.0) |
| Race |  |  |
| White | 109 (45.0) | 52 (45.2) |
| Black | 117 (48.4) | 60 (52.2) |
| Asian | 3 (1.2) | 1 (0.9) |
| Hispanic | 8 (3.3) | 5 (4.4) |
| Hospital |  |  |
| 1 | 89 (36.8) | 59 (51.3) |
| 2 | 153 (63.2) | 56 (48.7) |
| Flu Strain and Subtype/Lineage |  |  |
| Type A – H1 | 15 (6.2) | 44 (38.3) |
| Type A – H3 | 149 (61.6) | 47 (40.9) |
| Type B – Victoria | 2 (0.8) | 2 (1.7) |
| Type B – Yamagata | 62 (25.6) | 1 (0.9) |
| Unknown Subtype/Lineage | 13 (5.4) | 21 (18.3) |
| Unknown Type | 1 (0.4) | 0 (0.0) |
| Received Influenza Vaccine | 147 (60.7) | 73 (63.5) |
| **Comorbidities** | | |
| Charlson Comorbidity Index, mean (SD) | 3.1 (2.8) | 3.8 (2.9) |
| Charlson Comorbidity Index Group |  |  |
| 0 | 34 (14.1) | 13 (11.3) |
| 1-2 | 91 (37.6) | 35 (30.4) |
| ≥ 3 | 117 (48.4) | 67 (58.3) |
| BMI Category |  |  |
| Underweight (<18.5) | 7 (2.9) | 5 (4.4) |
| Normal/Healthy weight (18.5-24.9) | 51 (21.1) | 24 (20.9) |
| Overweight (25-29.9) | 59 (24.4) | 26 (22.6) |
| Obese (30-39.9) | 96 (39.7) | 40 (34.8) |
| Morbidly obese (≥40) | 29 (12.0) | 20 (17.4) |
| High-Risk Comorbidities |  |  |
| Heart Disease | 109 (45.0) | 61 (53.0) |
| Heart Failure | 58 (24.0) | 34 (29.6) |
| Asthma | 72 (29.8) | 27 (23.5) |
| COPD | 83 (34.3) | 44 (38.3) |
| Other Lung Conditions | 82 (33.9) | 56 (48.7) |
| Diabetes | 86 (35.5) | 54 (47.0) |
| Renal | 91 (37.6) | 51 (44.4) |
| Blood Disorders | 22 (9.1) | 15 (13.0) |
| Immunosuppression | 60 (24.8) | 35 (30.4) |
| Malignancy | 46 (19.0) | 29 (25.2) |
| Metabolic Disorders | 108 (44.6) | 63 (54.8) |
| Liver Disorders | 28 (11.6) | 10 (8.7) |
| Neurological/Musculoskeletal | 61 (25.2) | 37 (32.2) |
| Cerebrovascular Disorders | 10 (4.1) | 4 (3.5) |
| Endocrine | 41 (16.9) | 21 (18.3) |
| Long-term Medication | 35 (14.5) | 25 (21.7) |
| Morbid Obesity | 34 (14.1) | 24 (20.9) |
| **Outcomes** | | |
| ICU Admission | 15 (6.2) | 15 (13.0) |
| Mechanical Ventilator use | 24 (9.9) | 19 (16.5) |
| Hospital LOS, mean (SD) | 3.2 (2.9) | 3.6 (3.8) |
| Prolonged LOS (≥8 days) | 15 (6.2) | 9 (7.8) |

Data are presented as either column frequency (percentage) or mean (standard deviation (SD)), as appropriate. Missing data for physiologic and laboratory characteristics were imputed based on age- and hospital-specific mean values.

**Table S3.** Patient characteristics included in k-medoids algorithm overall by influenza season for exploratory analysis with statistically defined outliers.

|  | **2017/2018 Season** | | | **2018/2019 Season** | | |
| --- | --- | --- | --- | --- | --- | --- |
|  | **Full Data**  **(N = 242)** | **Imputed Outliers**  **(N = 242)** | **Number of patients with outliers** | **Full Data**  **(N = 115)** | **Imputed Outliers**  **(N = 115)** | **Number of patients with outliers** |
| **Clustering Metrics Measured Within 24 Hours of Admission** | | | | | | |
| Time from symptom onset to admission (days) | 2.4 (2.1) | 2.4 (2.0) | 1 | 2.6 (2.0) | 2.5 (1.9) | 1 |
| Temperature |  |  |  |  |  |  |
| Min | 97.9 (0.6) | 97.9 (0.5) | 5 | 98.1 (0.6) | 98.0 (0.5) | 3 |
| Max | 100.0 (1.5) | 100.0 (1.5) | 0 | 100.1 (1.5) | 100.1 (1.5) | 0 |
| Heart Rate (Max) | 107.0 (18.3) | 106.8 (17.3) | 3 | 110.7 (19.2) | 109.3 (17.5) | 3 |
| Systolic Blood Pressure |  |  |  |  |  |  |
| Min | 111.5 (16.7) | 111.0 (16.1) | 2 | 105.1 (15.6) | 105.1 (15.6) | 0 |
| Max | 137.0 (30.3) | 137.0 (30.3) | 0 | 126.3 (32.3) | 125.5 (30.1) | 1 |
| Glucose |  |  |  |  |  |  |
| Min | 117.0 (49.1) | 105.4 (25.1) | 21 | 118.2(56.3) | 105.2 (26.7) | 10 |
| Max | 159.5 (83.5) | 137.5 (38.9) | 24 | 175.0 (112.4) | 154.3 (65.3) | 7 |
| Creatinine (Max) | 1.3 (1.6) | 1.0 (0.3) | 18 | 1.7 (2.1) | 1.1 (0.4) | 14 |
| Hematocrit (Min) | 35.6 (5.4) | 35.8 (5.0) | 4 | 35.6 (6.2) | 35.6 (6.2) | 0 |
| Sodium |  |  |  |  |  |  |
| Min | 135.7 (3.1) | 135.9 (2.8) | 4 | 135.3 (4.0) | 135.6 (3.1) | 2 |
| Max | 138.1 (3.3) | 138.2 (3.2) | 2 | 138.1 (3.9) | 138.0 (3.6) | 1 |
| White Blood Cells |  |  |  |  |  |  |
| Min | 6.5(3.7) | 6.1 (2.5) | 6 | 7.6 (8.4) | 6.6 (3.2) | 3 |
| Max | 8.6 (7.6) | 7.4 (2.5) | 18 | 10.2 (12.9) | 8.3 (3.7) | 6 |
| Platelets (Min) | 178.6 (67.2) | 174.0 (52.4) | 10 | 193.8(119.4) | 185.3 (74.7) | 1 |
| Respiratory Rate (Max) | 24.2(5.5) | 24.1 (5.3) | 1 | 26.2(8.2) | 25.3 (6.6) | 4 |
| Oxygen Saturation (Min) | 91.6 (4.4) | 92.2 (2.9) | 9 | 90.6 (5.0) | 91.2 (3.8) | 4 |
| Estimated glomerular filtration rate | 75.0 (39.8) | 71.3 (29.5) | 7 | 73.6 (40.8) | 72.6 (39.1) | 1 |

Data are presented as mean (standard deviation)

**A.**
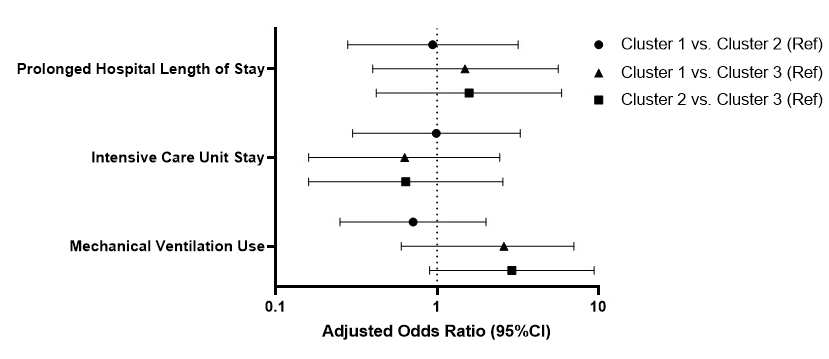
 **B.**
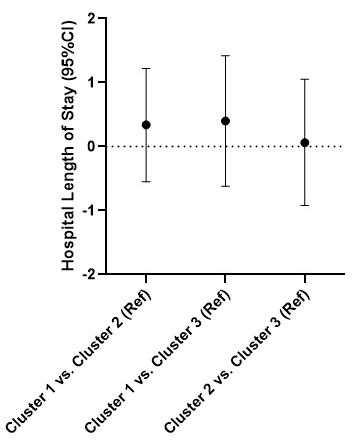


**2017/2018**

**C.**
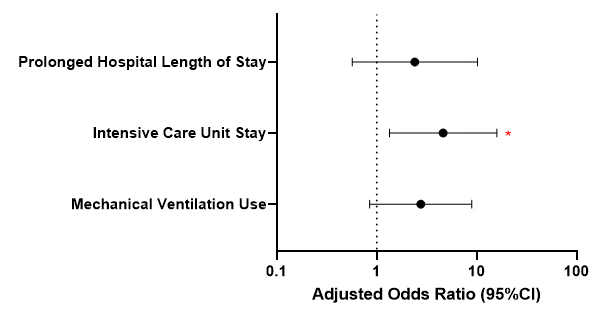
 **D.**
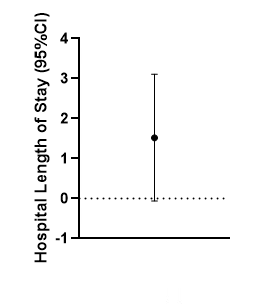


**2018/2019**

**Figure S1.** Adjusted odds ratios (A, C) and difference in model-adjusted means (B, D) with 95% confidence intervals for outcomes. Models were adjusted for age group, sex, hospital, continuous CCI, and influenza vaccination status.
